# How to Frame Destination Foodscapes? A Perspective of Mixed Food Experience

**DOI:** 10.3390/foods11121706

**Published:** 2022-06-10

**Authors:** Dan Zhu, Jiayi Wang, Peng Wang, Honggang Xu

**Affiliations:** 1 Glorious Sun School of Business & Management, Donghua University, Shanghai 200051, China; zhudan@dhu.edu.cn (D.Z.); 2201290@mail.dhu.edu.cn (J.W.); 2 School of Tourism Management, Sun Yat-sen University, Zhuhai 519000, China; xuhongg@mail.sysu.edu.cn

**Keywords:** food experience, foodscape, tourism destination, travelogue, netnography

## Abstract

Foodscape conceptualizes the dynamic human–food–place nexus. Tourism provides a cross-cultural context where tourists can consume different destination foods and places, during which multiple types of destination foodscapes are produced. However, few studies explore how to frame the types and connotations of destination foodscape. Tourists’ travelogues provide a rich database to examine this question. Through netnography, this study collects and analyzes 86 posts of travelogues published from 2012 to 2019 in Mafengwo, a famous Chinese online travel community, about Chinese tourists’ food experiences in Chiang Mai, Thailand. We summarize five types of destination foodscapes, globalized recreational foodscape, staged local foodscape, glocalized foodscape, authentic local foodscape, and overseas ethnic foodscape in which tourists obtain different familiar-novelty hybrid experiences. This study contributes to interdisciplinary dialogue between food and tourism literature by proposing a coordinate framework with two axes, the spectrum of cultural distance and the spectrum of serving tourists/locals, to classify destination foodscape and a six-dimensional network construct to reveal the connotations of destination foodscape. Relevant strategies for promoting destination food and tourism development are also provided.

## 1. Introduction

During the past two decades, the notion of foodscape has attracted wide academic attention in both social and natural sciences, including nutrition, health, urban geography, public policy, consumer studies, food sciences, etc. [1,2,3,4,5,6,7,8]. It is an aggregative concept interconnecting the place, identity, culture, foodstuff, service and human and provides scholars relational thinking about these actors. Specifically in the consumer and cultural studies, foodscape helps us understand how people consume and experience food in interaction with the environments and social-cultural contexts. They connect with each other to form a complete gastronomic experiencescape [7].

In a tourism destination, food is one of the core elements for tourists to experience the destination’s attractiveness and obtain an unforgettable memory. Previous food tourism literature has gone through three stages from the producer-oriented (investigate the destination food itself) to co-creative (tourists’ involvement in destination food consumption) and then to the holistic and relational perspective (foodscape) [7]. In this process, a contradictory nexus exists between destination food and tourists. Tourists, to different degrees, are eager to experience novel and exotic tastes during travel. At the same time, the domestic stomach and diet habits may constantly interrupt them from really enjoying the divergent food for too long [9,10,11,12]. Hence, tourists would be more likely to seek a balance between the exotic and familiar, leading them to step in the direction of seeking proper foods in this cross-cultural interface. Previous scholars have explored some modes of tourists’ food consumption to solve this contradiction, such as eating fast food or buying local ingredients but cooking by themselves [12,13].

However, in this process, not only the food itself but also the physical settings, dining etiquettes, human interactions, and atmospheres all express different familiar and novel stories. They are linked and experienced by tourists. Thus, along with the encounters between different destinations and tourist-generating cultures, multifarious foodscapes with multiple mixed experiences are created [14,15]. Then, an in-depth investigation into framing the types and connotations of destination foodscapes in specific contexts is warranted. It could reveal how tourists, destination places and foodstuffs interact as a network to produce a satisfying food experience, thus benefiting the destination gastronomic industry and tourism development.

The travelogs posted by tourists online provide a rich database to explore this question. The Web 2.0 era endow individual tourists a chance to save and share their food memory during a trip by publishing their photographs and travel notes on the online tourism agencies (OTA) social media anywhere with the help of a computer/smartphone and the internet [16,17]. The pictures and words they post represent their gaze of the destination, their embodied experiences of certain foods, and their emotional feelings on-site. They elaborately select pictures and write down notes as an intuitive way to relay the food experience and assemble the destination foodscapes for readers, thus becoming a bridge for cross-cultural understandings and providing e-WOM (e-word of mouth) to the public [18]. However, the current literature has relatively neglected performing detailed work in this digital area to track the production and characteristics of destination foodscapes.

Hence, through a netnography of mainland Chinese tourists’ travelogs about their food experience in Chiang Mai, Thailand, this paper aims to answer two questions: (1) How do we classify the destination foodscapes from the perspective of tourists’ mixed food experience? (2) What specific dimensions and connotations of destination foodscape can be extracted? Our paper is structured as follows: We first briefly review the studies about foodscape and then move to the tourism literature. By emphasizing the characteristics of the tourism context, we would elaborate on the destination foodscape as a kind of mixed landscape. Then, we review the connections between food tourism and the online virtual community. This is followed by the study context and methods. In the findings section, a total of five different foodscapes will be elaborated on one by one. The final section is the conclusion and discussion section, highlighting the typological framework and the dimensional construct of destination foodscapes as our main contributions to food and tourism studies.

## 2. Literature Review

### 2.1. Foodscape: A Brief Review

We live in a world surrounded by food and meals. The concept of foodscape describes this complex social system where humans interact with the foodstuff and place through various practices [7,14,19]. It is composed of “food” and the suffix “-scape”, which shows an advantage in studying the food-related phenomena that are unevenly distributed in space and contexts [20]. Hall and Gössling emphasized that foodscape is a dynamic concept as it reveals a changing picture of how food is embedded and connected with the exterior physical, social, perspectival and cultural surroundings [21]. According to different geographical scales, the foodscape ranges from the microscopic (e.g., body, kitchen) to the meso level (e.g., community, region) and the macro level (e.g., nation) [3].

After around 20 years of knowledge accumulation, the current foodscape studies can be generally divided into three primary research directions. First is foodscape in the sphere of nutrition and public health studies. This direction aims to promote more nutrition-friendly foodscapes and improve public health [22,23,24,25]. Second is the construction of ethical, equitable and sustainable foodscapes, mainly in the spheres of politics, geography and sociology [26,27,28,29,30,31]. The third is the foodscape consumption and experience, mainly in the consumer behavior and culture sphere [2,32,33,34,35]. The three directions of foodscape studies overlap with different academic emphases. A consensus exists that this concept is useful for approaching a holistic and relational understanding of the complex network composed of humans, food materials, the environment and culture [6,8,19].

Specifically, in the third direction of foodscape consumption and experience, culture is highlighted as a significant and dynamic variable in influencing consumers’ experience and the construction of foodscapes [3,8]. Unlike the habitual daily life that is paid attention to by the majority of studies, tourism provides a cross-cultural context where destination food acts simultaneously as a tourism attraction and a supporting necessity for tourists [11,12]. In this sense, the destination foodscape can be seen as an interactive and co-created landscape among tourists, food and destination places. Then, how do we conceptualize the destination foodscape considering the unique cultural encounters tourism creates? What are its connections with tourists’ food experiences? The answers to these questions are quite important as they provide a rethinking of the conceptualizations of foodscape and food consumption in current literature. However, they are relatively ignored by current scholars.

### 2.2. Foodscape in Tourism: A Mixed Landscape

The most crucial distinction of destination foodscape from those in daily contexts is its mixed feature in the cross-cultural interface tourism brings. On the one hand, tourists leave their homes to seek an exceptional destination landscape. Foods become one of the core attractions to mark the destination’s uniqueness and create memorable experiences [36,37,38]. Tourists seek novelty not only from tasting the food and learning about the ingredients but also from the embodied experience of the tangible and intangible dining environment, the identity and interactions with the service staff, other guests and locals [33,39,40]. On the other hand, tourists also need food to satisfy their hunger needs and replenish themselves [13,41]. In this sense, eating becomes a habitual and supporting activity [10]. Tourists seek familiarity, comfort, and ontological security from the destination food and the related places to deal with possible physical and psychological maladjustment [42]. For example, previous studies have found that when Chinese tourists visit Europe, even though the taste and environment are not that authentic, they would more or less choose the local Chinese restaurant because of their discomfort with the local food and eating habits [11,12,13,43]. Hence, pushed by these two opposite motives, tourists with different novelty tolerance would search for different destination foods and dining environments. In this process, tourists connect different destination materials and social-cultural actors, and various types of foodscapes are being produced. The destination foodscapes can thus be seen as a kind of mixed landscape hybridized with local and global, indigenous and alien, traditional and modern, and host and guest cultures.

Current tourism studies mainly proposed four models to describe tourists’ food choices in destination, which provide a useful line of thought to frame the different types of destination foodscapes. The novelty-familiarity binary structure [10,41], the core-periphery structure [9], the novelty-familiarity spectrum model [13,15], and the two-dimensional matrix model [14]. The novelty-familiarity spectrum model is developed based on the binary and core-periphery structure to argue for more mixability and in-betweenness of tourists’ food consumption and experiences. Lin et al. summarized four types of destination foods: a destination’s local food, overseas Chinese food, global fast food, and tourists’ home food [13]. It deserves further consideration of the embeddedness of these foods in the destination context and their possible assemblages of foodscapes. The two-dimensional matrix model proposed by Björk and Kauppinen-Räisänen is mainly based on the dimensions of the organized/unorganized environment and the stage for tourists/locals [14]. Based on it, they summarized four types of destination foodscapes: destination service encounter, local service encounter, destination encounter, and local encounter. However, this model relatively ignores the dimension of culture in tourism encounters.

As a holistic and abstract concept, the inner dimensions and connotations of destination foodscape also need more research attention. Currently, Björk and Kauppinen-Räisänen have summarized five dimensions: physical environment, social interactions, food quality value, monetary value, and divergence [14]. The physical environment includes the physical location, the décor, the functionality, and the service encounter’s story. Social interactions include the interactions with (or the immersions among) the service staff, the guests and the family members. Food quality values include food sensation and food locality. Monetary value refers to the price–quality relationship. Divergence refers to the difference or special happenings. The independent dimension of “divergence” indicates an emphasis on the exceptional and novel features of tourists’ food experience, ignoring the novelty-familiarity mixed feature that tourists may obtain from the other four dimensions. In addition, as this construct is mainly referred to from the servicescape perspective, the element of culture in the connotation of destination foodscape lacks full consideration.

### 2.3. Posting Travelogs: Assembling Destination Foodscape Online

As tourists use online blogs and social media to plan and record their travel experiences globally, a vast virtual community emerges [17,44,45]. Tourists use their accounts of online tourism agencies or various social media apps to post their travelogues in texts, photographs and videos. As food opens a wonderful journey of visual, taste and emotional senses and fixes a deep memory of the destination place, the food experience is frequently recorded by tourists and receives a large audience [16]. In this virtual community, tourists with pseudonyms have high freedom to express and present what they see, think and feel with the destination food [46,47]. As there is no clear target to satisfy a specific audience, tourists can flexibly entertain themselves by presenting fragrant and colorful dining images, with the texts narrating their authentic experiences [48]. Hence, in a travelogue, the tourist’s narration of their on-site experience connects a relational network of the destination food, the physical environment and ambiance, the service and human interaction as a way to perform the destination foodscape.

Hence, compared with real society, the virtual community of online travelogues provides a secondary world for tourists to record and assemble the destination foodscape, providing a rich database to research how different destination foodscapes are performed through the perspective of the tourist food experience. However, it is still ignored in the tourism literature, just as Okumus has argued that more scholarly attention should be paid to the technology and media and their connections with food experiences and culinary destinations [49].

## 3. Study Context

The travelogues of Chinese tourists about their food experience in Chiang Mai, Thailand, were chosen as our study case. It was chosen for three reasons. First, as an international tourism host country, Thai food is well-known worldwide. A recent ranking of CNN travel put Thai Massaman curry and Tom Yam Kung into the top 10 best foods [50]. Chiang Mai is a famous destination city in Northern Thailand, attracting international visitors with its diverse food offerings [51,52]. The foods are embedded in different forms such as local markets, restaurants, street stalls, cooking schools, and even convenience stores [53]. Thus, Chiang Mai provides a typical destination case with multiple food resources for tourists to discover and consume.

Second, before COVID-19, mainland Chinese tourists had been one of Chiang Mai’s biggest tourist-generating markets. In 2012, a Chinese comedy film, “Lost in Thailand”, was released. It promoted Chiang Mai to be a famous outbound destination city for Chinese mass tourists [54,55]. China and Thailand are neighboring countries, both belonging to the Asian cultural circle. When Chinese tourists visit Chiang Mai, the cultural distance and cultural affinity existing in this encounter interface make it possible for tourists to seek foods between novelty and familiarity. Therefore, it provides an interesting context to explore how international tourists deal with the local and global, the indigenous and alien elements and engage in constructing different foodscapes.

Third, the first author has conducted research on Chinese outbound tourism to Thailand for more than five years and has conducted field research twice in Thailand. One was conducted in Chiang Mai, from 8 to 15 August 2019, to investigate Chinese tourists’ behavior and experience. During that week, the first author visited and tried different foods in different settings, including night markets, restaurants, malls, and so on, within the old towns and suburbs. This experience gave her an “insider” perspective to understand the destination context, which is helpful for the formal data collection and analysis of tourists’ digital travelogues [16].

## 4. Method

### 4.1. Data Collection

This study aims to frame the typology and connotations of the destination foodscapes through the perspective of tourists’ experiences. Based on previous fieldwork, netnography is adopted to collect and analyze tourists’ travelogues about the destination food experience. There are two characteristics of netnography highlighted by Marotzki: the use of qualitative methodological tools and primarily concerning the online community [56]. It is developed from ethnography, emphasizing a naturalistic approach to describing and interpreting cultural phenomena [57,58]. Along with its wide use in consumer culture, business and tourism studies, netnography gradually shifts to adapt to the wider research questions of these disciplines [59,60,61,62]. The isolated personal reviews, blogs and communications and the interactions and public discussions of online communities can both be evaluated by netnography to reveal the cultural phenomenon related to market, consumers, places, etc. Netnography demands a more succinct research agenda with flexible requirements in researchers’ length of stay and participative interaction within the online community, according to the change in research questions [62].

Mafengwo was chosen as the online tourism community where we collect tourists’ travelogues about their food experience in Chiang Mai. It is one of the Chinese leading online tourism agencies (OTA) providing high-quality travelogues publicly. Compared with another famous Chinese OTA, “Ctrip”, the travelogues in Mafengwo are less in number but more detailed and richer in content and length, which can help us understand the context and conduct thick and grounded data analysis required for netnography [57]. After full reading and familiarity of tourists’ travelogues about Chiang Mai in Mafengwo, the data collection was formally conducted during 1–11 April 2022. The first two authors collected 93 travelogues by searching the keywords “Chiang Mai” and “food” (in Chinese) in the search engine Mafengwo. Then they read the travelogues one by one, deleted those unrelated to Chiang Mai food experiences and obtained 86 travelogues (mainly containing texts and photos) from 2012 to 2019 (see Table A1). These constitute the “archival data”, meaning the direct data from the Web, not a product of the researcher’s involvement in creating the data [57] (p. 266). To further understand the context and tourists’ subjective experiences, we made efforts to collect “elicited data”, the data co-created by the researcher and members of the social media community through social interaction [57] (p. 267). From 11 to 17 April 2022, we contacted these travelogue authors by sending them messages and finally obtained four informed consents. Three of them were interviewed by us through the software Tecent Meeting and another through WeChat messages (see Table A2). During the whole process, we kept recording field notes about the in-the-moment reflections on Chiang Mai’s foodscape. The different phases of data collection contribute to its triangulation, thereby improving the reliability of this study [63].

The ethics are fully considered. As Mafengwo is a public community for tourists to post their travelogues and for visitors to read, it is unnecessary to obtain permission from the bloggers to collect and analyze these UGC (user-generated content) data [64,65]. However, when collecting interviews with the travelogue bloggers and quoting and illustrating their photos, we all sent the request and obtained their informed consent [57,66]. Even though most bloggers use pseudonyms, all the travelogues are anonymously numbered in Arabic numerals to strengthen the protection of bloggers’ privacy.

### 4.2. Data Analysis

**Figure 1:**
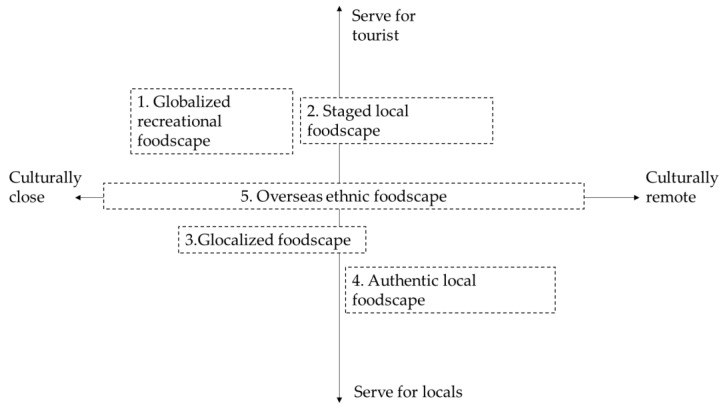
Five types of destination foodscapes in the coordinate typological framework of destination foodscape.

The data analysis was conducted synchronously. It consists of four stages. First, the first two authors carefully read all the travelogues back and forth to understand the tourism context. Second, they selected the content directly related to tourists’ food experience in Chiang Mai and copied them into new Word documents. Third, they read the contents again and manually conducted coding for thematic analysis. Coding is a common tactic for analyzing qualitative data in netnography [16,57,67]. The first two authors mainly conducted the coding on both the pictures and texts, and they kept discussing with the other two authors to improve the coding trustworthiness [68,69]. Through an inductive and iterative approach, the authors conducted coding with a repetitive conversation regarding previous literature. We mainly referred to the dimensional framework of destination foodscape proposed by Björk and Kauppinen-Räisänen [14]. We compared the data with their dimensions, modifying them or constructing new dimensions in this process. Finally, a six-dimensional construct of destination foodscape emerged, including consumption grade, physical environment, social interaction and ambiance, food and eating, culture and tourist mixed experience. By engaging with these dimensions, tourists obtain different patterns of mixed experiences. Our coding generally reveals five patterns of tourist experience, corresponding to the emergence of five types of destination foodscapes. They are represented in the coordinate system of “culturally close-remote” and “serving tourists-locals”, namely, globalized recreational foodscape, staged local foodscape, glocalized foodscape, authentic local foodscape and overseas ethnic foodscape (see Figure 1). Next, in the findings section, we will elaborate on the five types of foodscapes one by one with illustrative quotes from the raw data.

## 5. Findings

### 5.1. Globalized Recreational Foodscape

The globalized recreational foodscape is culturally affinitive and provides considerate services to international tourists, with recreational ambiance. The Nimmanhaemin Road in Chiang Mai is a representative case. It is located between the Chiang Mai ancient town and Chiang Mai University. It was initially a residential area consisting of several crossing streets. The increase in international tourists attracts capital investments, and many creative eateries for recreation are open to tourists at medium to high-end price levels.

**Figure 2:**
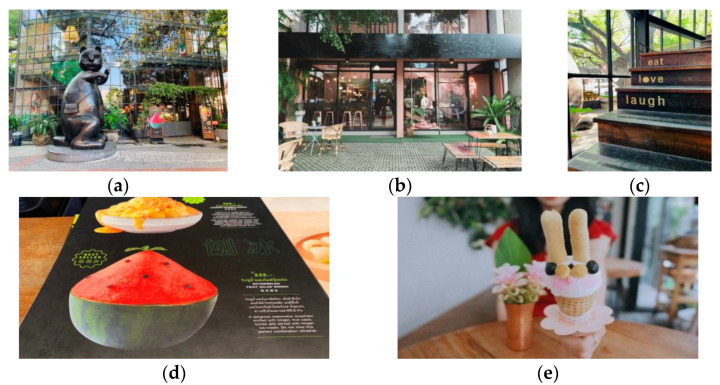
Images of globalized recreational foodscape: (**a**–**c**) the modern facade with lovely cartoon models; (**d**) the menu with Thai, English, and Chinese languages; (**e**) tourists taking photos with the creative and lovely desserts used with permission (this applies to Figure 3, Figure 4, Figure 5 and Figure 6).

The facade of the shops and restaurants in this area is generally in a modern style but with various decorations. Tourists’ travel notes depict a colorful, bold, free and whimsical creative block (Figure 2a–c). Tourists always stroll and spend their lazy afternoons and nights here, expressing a relaxed and comfortable feeling.

*Wandering around Nimmanhaemin Road all afternoon, the sun is hot and the mood is slow. The pressure at work and the tense rhythm on weekdays are temporarily away. It is a significant benefit of travel.*

*(M84)*

*Nimmanhaemin Road is nothing special, but there are full of cafes, restaurants and small shops on these staggering paths. Many of these small shops are operated by local artists. The decoration and style of these shops seem to be a platform to show their talents. The petty bourgeoisie and fresh emotions of Nimmanhaemin Road come from these businesses.*

*(M73)*

The modern and globalized fashionable shops along the roads turn this area into a tourist clave. Tourists visit and consume the drinks, desserts and meals here, with little interaction with the locals or other tourists. The logos and menu texts are mainly Thai, English, and Chinese (Figure 2d). Hence, tourists stay within the environmental bubble, take photos and watch visitors come and go. As tourists write in their travelogues, *“Listening to Thai folk songs, drinking coffee and watching passers-by are the best choices to kill time. (M73)”, “Sit and watch the waves of tourists coming to tick off the shop and take pictures (M72)”.*

These recreational eateries are popular for providing globalized drinks and desserts such as coffee and ice cream. Hence, the flavors are mostly familiar to tourists worldwide. The local fresh fruits such as watermelon, mango and coconut are the main local ingredients used to make the desserts. Although the ingredients and tastes are familiar, most tourists are amazed by the lovely and creative appearances of the desserts and drinks. They describe the dessert-eating as a” treasure hunt (M16)” process and take many photos with them here (Figure 2e).

Therefore, the collection of international popular desserts and cafeterias mainly caters to tourists with a minimized cultural shock. Most times, tourists stay in the environment bubble and enjoy the mother-tongue language service and the familiar casual foods. There is a minor extent of novelty reflected in the artistic decorations, creative appearances of the food, the Thai serving staff standing by and the international ambiance with tourists coming and going.

### 5.2. Staged Local Foodscape

Our analysis also reveals a ubiquitous type of “staged local foodscape”, which mainly caters to international tourists’ curiosity about Thai cuisine culture. Representatives of this type of foodscape are the Thai cooking schools and Thai-style restaurants that international tourists especially consume, with many Thai symbols offered on stage. The prices also range from medium to high grade.

**Figure 3:**
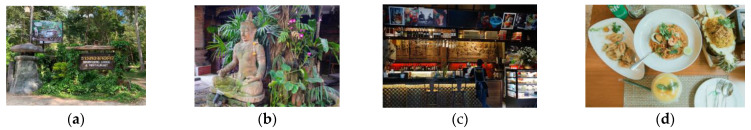
Images of staged local foodscape: (**a**–**c**) the physical environment with Northern Thai symbolical tropical environment and Buddhism culture; (**d**) the typical local Thai dishes with a combined flavor of western food.

The cooking schools and Thai restaurants pay great attention to presenting the “local” elements in the physical environment. Reflected from tourists’ travel notes, the local Lanna culture, the Theravada Buddhism and the tropical vegetation garden environment are the representative local images staged in these spaces and gazed upon by tourists (Figure 3a–c). Hence, most tourists express their deep visual impressions in travelogues:

*“The walls are full of Buddhist statues, very Thai-style.”*

*(M01)*

*“Tharnthong restaurant is a “secret garden” hidden in the jungle. The restaurant is surrounded by a wide range of green plants and gurgling streams, a scattered layout. It is resolutely a Thai version of a private garden...”*

*(M16)*

The service personnel can always speak the tourists’ native language to smooth the tourists’ understanding. For example, tourists follow the teacher in the Thai cooking school and learn to cook three to four representative Thai dishes, such as Tom Yum Goong, green papaya salad, Pad Thai and curry chicken. Although with nonstandard pronunciation, the Thai teacher can speak English or Chinese to fulfill the tourists’ understanding. There are sometimes several groups of international tourists from different nationalities, but they rarely communicate with each other. Relatively, self-cooking, taking photos and feeling the exotic ambiance are enough to meet these tourists’ needs for novelty.

*“Open fire! Then my stove suddenly failed to open fire. I said, great. I’m here to take photos. I’m more interested in taking photos than cooking. Later, most of my dishes were cooked with the help of my husband.”*

*(M20)*

Concerning the food and eating experiences, tourists all emphasize enjoying the local fresh ingredients and dining in the Thai-style ecological or cultural settings. However, most tourists are likely to search for a palatable rather than an objective authentic taste of Thai food. To cater to the tourists’ taste, the cooking school or the restaurant has some flexibility to reform the local Thai flavor. For example, in the famous garden restaurant Chom Café and Restaurant, Thai ingredients are combined with some popular Western food styles (see the curry spaghetti in Figure 3d). In the Thai cooking school, the tourists also choose what and how much seasoning to use.

*“Besides listening to the teacher’s explanation, you could also consider your tolerance. Don’t be greedy to put peppers and lemons. Otherwise, only eat with crying.”*

*(M02)*

Hence, corresponding with the findings of Walter, this staged local foodscape is created by the force of tourists’ superficial pursuit of local culture [16]. On the one hand, they hope to collect the local Thai symbols by photographing the Northern Thai-style dining environments and tasting typical dishes. On the other hand, most of them still stay in a familiar “environmental bubble” as they enjoy the reformed taste of the local food and a comfortable and familiar dining environment with exclusive services offered to them.

### 5.3. Glocalized Foodscape

Some international chain restaurants and stores scattered in Chiang Mai, such as the seven-eleven convenience store, the McDonald’s, KFC, and Starbucks, are open to locals. They also attract international tourists by bringing tourists a standard price and familiar space to eat with temporary comfort, just as M10 expresses, “*I can’t help but enter as long as I see the seven-eleven convenience store*”.

The physical environment of these dining spaces is decorated following a uniform standard. Tourists all express that it is pretty easy to recognize their distinctive logos on the facade. However, these global chain restaurants also take some localized form in decoration. For example, some tourists take photos with the Thai McDonald statue. It shows a very Thai-style welcoming gesture of putting the palms together, giving tourists a sense of exoticism. As M38 writes, *“I must take a photo as it’s McDonald’s unique pose in Thailand. (M38)”*

**Figure 4:**
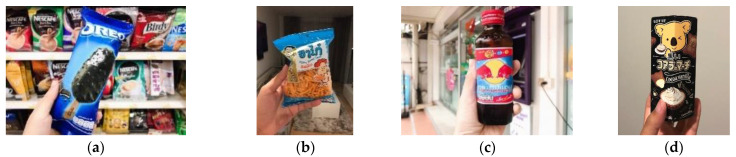
Images of glocalized foodscape: some snacks with global brands, (**a**) Oreo ice-cream; (**b**) puffing food; (**c**) the Redbull drinks; (**d**) sandwich biscuit.

Tourists’ travel notes and pictures reveal that many residents eat and go shopping in this type of space, indicating their gaze upon the locals. Tourists also show great interest in the food, snacks and desserts in these eateries. They found that most of them are in standard packages, brands and tastes, similar to those in their native country, such as the Nescafé, the Redbull drinks, the milk, and yogurt (Figure 4). Some tourists make fun of them by saying that *“These foodstuffs save us. I sighed for many times that it’s too happy to be a clerk of seven-eleven in Thailand! (M36)”*. However, tourists also noted some “glocalized” food made from the local ingredients that they cannot source at home, with a surprising flavor.

*“In particular, KFC on the fourth floor of Maya has large cups of mashed potatoes, pineapple-flavored cones and beautiful soda that we can’t eat in China.”*

*(M02)*

*“I ate corn pie at McDonald’s; milk flavored and not available in China. Yummy~~.”*

*(M35)*

Therefore, even though these restaurants and stores have not provided unique and exclusive services to international tourists, their globalized logos, decorations, and familiar food packages and formula shorten their cultural distance with tourists in a foreign country and thus still attract tourists’ visitation and consumption. Tourists enjoy the locals’ eating ambiance and are also happy to try some glocalized flavors. In this sense, this dining trip is carried out within a familiar environmental bubble hybrid with some local novelty, hence the name of the glocalized foodscape.

### 5.4. Authentic Local Foodscape

The authentic local foodscape refers to what Edensor called the “heterogeneous space” [70] (p. 327), where tourists seek the local foods. It is the space where locals live, produce and consume foods, and heterogenous encounters between different people could happen. The night markets and restaurants serving the locals are typical of this type of foodscape. They are generally cheap at the folk level of consumption. Tourists intrude these local spaces with a solid curiosity to experience the local mundane food and life:

*“I think when travelling, including the domestic travel, I must not go to that kind of place crowded with tourists. Well, I think this kind of place will sometimes, for example, in order to cater to tourists, um, its taste, or its price is not that particularly reasonable.”*

*(I01)*

**Figure 5:**
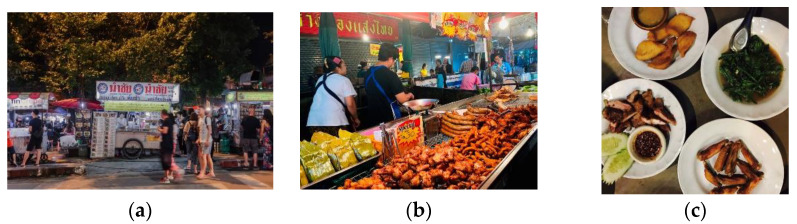
Images of local Thai foodscape: (**a**) the street vendors; (**b**) locals sell and buy fried meat; (**c**) some favorable local dishes adaptable to the taste of Chinese tourists.

In terms of the physical environment, the local night market is usually in the form of mobile street stalls along the road (Figure 5a). The restaurants are also humble, without special decorations, “just *as the food stalls in China, low grade and cheap (I02)*”. Hence, there is no high-standard management in their hygienic conditions. Most of the foods are labeled in Thai. Tourists have to communicate with the locals by finger gestures or showing photos. Some tourists recalled their unique experience of dish orders in the local restaurants:

*This small shop of pottery hot pot is located in the snack street at the back door of Chiang Mai University. When I went there, it was time for dinner. It is full of university students eating the hot pot with glutinous rice. The menu is only in Thai, and the order is handwritten by oneself. Therefore, we can only take pictures of what the students eat and show them to the landlady to finish the dish order.*

*(M02)*

*There is only Thai and no English in most authentic Thai snack stalls. Sometimes it’s very embarrassing as I don’t know how to order the food. I could only watch what others buy first and then communicate with the shopkeeper who can’t speak English.*

*(M04)*

Therefore, interactions frequently happen between tourists and locals. Tourists also have a chance to observe the interactions among locals (Figure 5b). Such a lively and exotic stage endows tourists in moving out of their environmental bubble to experience the authentic local culture. It corresponds with what Björka and Kauppinen-Räisänen depict as “immersion” [14]. Tourists immerse into the local mundane lives as a component of their food experience.

Concerning the taste and quality of food, tourists’ travel notes present different ideas. Some can adapt to the local sour and spicy taste, but some cannot. Generally, we find that Chinese tourists present gradually changing adaptations to the food in this local Thai foodscape. Most Chinese tourists can accept and enjoy those foods close to the diet in Southern China, such as the Hainan chicken rice, Chaoshan pig feet rice, and charcoal roast pig neck meat (Figure 5c), but show diversified adaptations to some of the traditional dishes such as the thick curry and some local herb condiments of Northern Thailand:

*“Thai food is unforgettable on the first try because of its solid and stimulating sour, hot, and sweet taste. People who love enjoy the flavors mixed with all kinds of spices. People who hate taste it once and never want to touch it again.”*

*(M02)*

This type of foodscape provides tourists with a strong sense of authentic flavor due to the unorganized physical settings and heterogenous contacts among locals and between locals and tourists.

### 5.5. Overseas Ethnic Foodscape

**Figure 6:**
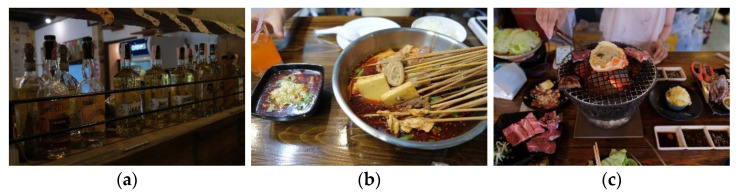
Typical images of overseas ethnic foodscape: (**a**) the interior setting of a Mexican restaurant; (**b**) the spicy food of a Chinese Szechuan restaurant; (**c**) a restaurant of Japanese barbecue.

Another particular foodscape, the overseas ethnic foodscape, provides a choice for tourists beyond the local and global food. The ethnic restaurants of different countries, e.g., China, Italy, India, and Japan, represent this type of foodscape (Figure 6). They present a more diverse distribution in the spectrum of cultural distance. Tourists visit them either to seek their familiar domestic food or *“change a taste (M85)”*. Our data also reveal that these overseas ethnic restaurants are inclusive for both the locals and tourists at the medium to high level of consumption. These determine its location in the coordinate system (Figure 1).

Tourists show a particular interest in capturing the national cultural symbols from the physical decorations and the layouts of these ethnic restaurants. For example, when writing about their dining experience in the Yangzi Jiang Cantonese Cuisine, M85 posted a photo of a Chinese calligraphy “Le” hanging on the wall of the restaurant with the words: “*The interior setting is also completely Chinese style.“ (M85)* To them, the mother tongue characters and some typical national symbols in the physical setting immediately bring a sense of intimacy and familiarity. As M81 noted, *“It must be the Chinese words on the wall that attract us. It’s very nice to see them in a foreign country” (M81).*

The interactions between tourists and the service staff are also limited to ordering food. Tourists usually gaze upon the local Thai people dining in these overseas ethnic restaurants. Just as M85 is quite proud to share that she met the Thai locals in a Chinese Yunnan restaurant:

*“I saw a big Thai family, a total of 13 family members, take their pickup trucks and come to this restaurant to eat. It can be seen that this restaurant is quite famous among the local people.”*

*(M85)*

Comparatively, tourists care more about the taste of these ethnic foods. Many tourists mention that they need Chinese food to save their stomachs and even *“renew the life (M36)”* on a trip. Therefore, they engage in a dining trip to search for their familiar tastes. Sometimes they find that some dishes are reformed by some Thai cooking styles, which can be seen as a familiar experience hybrid with a small amount of locality. As I01 describes, “*The store played Chinese songs popular in TikTok* *and we ate the skewered food served in hot pot. The taste was not as authentic as in China. (I01)*” Sometimes tourists also choose some foreign ethnic restaurants to change their tastes. These foreign ethnic foodscapes diversify their dining experiences in Chiang Mai with a sense of surprise, which can be indicated by this tourist’s words:

*“Tonight’s feast is to experience Indian cuisine. The unique charm of Chiang Mai lies in the authentic cuisine of all countries…I feel good when I eat with my hands and the Indian pancake is delicious.*

*(M63)*

Hence, tourists choose these overseas ethnic restaurants to diversify their diet and taste and obtain different mixed dining experiences. In the mother-country ethnic restaurants, tourists mainly obtained comfortable and familiar experiences, with a temporary return to ontological security. However, along with the aggravation of cultural distance, tourists renew their exotic experience in those foreign ethnic restaurants in Chiang Mai.

## 6. Conclusions and Discussion

### 6.1. Research Summary

Tourism creates a cross-cultural interface where food becomes a significant actor in connecting tourists with the destination and brings about diversified experiences. Eating food at destinations can be a supporting activity to satisfy hunger needs and keep ontological security or a novel experience to taste new cultures. In the food consuming and eating process, tourists are not only connected with food but also gain perspective and obtain sensory connections with the destination places. Thus, they are networked and assembled as a synthesized landscape and the destination foodscape, providing a broad vision of improving destination attractiveness [7]. Tourists publish their food experiences through descriptive words and lively visual materials, providing a rich database to explore the multifarious destination foodscapes. Through thematic analysis of Chinese tourists’ travelogues about their food experience in Chiang Mai, Thailand, this study investigates a framework to classify different types of destination foodscapes and develop the dimensional construct of destination foodscape.

**Figure 7:**
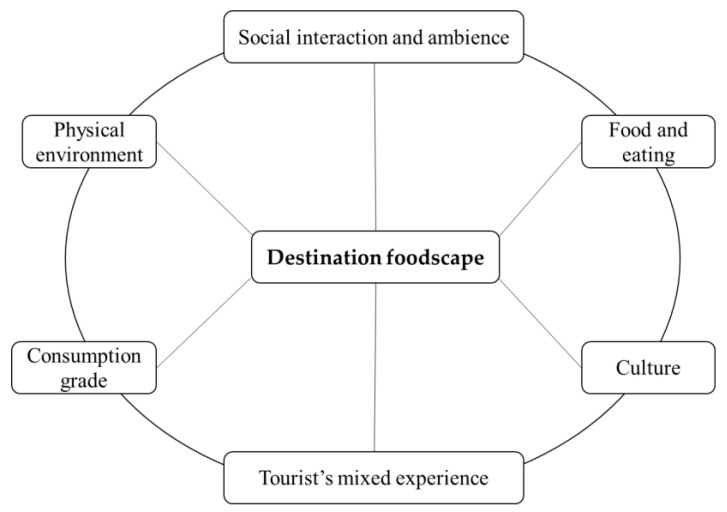
The dimensional construct of destination foodscape.

We found five types of destination foodscapes located differently in a two-axis coordinate system. The two axes respectively refer to its cultural distance away from tourists (from close to remote) and the primary market it serves (from tourists to locals). The closer the cultural distance and the more exclusive service for tourists, the more familiarity tourists will obtain. Based on the dimensional construct developed by Björk and Kauppinen-Räisänen [14], we refined six interrelated dimensions depicting the connotations of destination foodscape through our raw data. They are consumption grade, physical environment, social interaction and ambiance, food and eating, culture and tourist’s mixed experience (Figure 7). Table 1 summarizes the features of the five destination foodscapes in these dimensions.

The consumption grade refers to the price level and grade tourists pay to eat in a particular type of foodscape. Although it is not a direct factor causing tourists’ familiar or novel experience, it is firmly correlated with the service exclusiveness, the physical decoration and the food-making methods in the foodscape, which further express different, familiar or exotic stories to tourists. The physical environment includes the dining space’s functionality, location, and decoration, which stimulates tourists’ sensory, emotional and imaginary feelings connected with familiarity or novelty. Social interaction and ambiance include the possible interaction between tourists and the locals, service personnel or other guests, or the feeling of the social ambiance in the foodscape. Tourists can obtain different familiar–novel hybrid experiences from different social interactions or ambiances. Food and eating include multiple elements such as food ingredients, taste, cooking style, hygiene, healthy effect, appearance, eating method or dining habits. It is the core dimension bringing tourists familiar or novel experiences. Culture is a relatively implicit dimension in the destination food that tourists will sense in their embodied dining practices. Our findings indicate that globalized food culture is more familiar to tourists than the destination cultures, along with the globalization of capital. The overseas ethnic restaurants present different national cultures according to their ethnic country. However, the global, local and ethnic cultures do not independently appear but are always hybrid with each other in different types of foodscapes, bringing tourists diverse encounters of mixed cultures. The final dimension is the tourist’s mixed experience, which can be seen as an outcome of tourists’ engagement and networking with all the former dimensions.

It should be noted that departing from different social and demographical backgrounds, different tourist groups may encounter disparate familiar–novel experiences from the former five dimensions. Thus, a specific dining space might be sorted into different types of foodscapes by different tourists. An example from our data is the night markets in Chiang Mai. The first-time visitors quickly feel exotic about the destination materials and places. Hence, they always choose the weekend night markets that are specialized for the international tourists, with English and Chinese signs and labels everywhere, as the authentic local foodscape. However, to those tourists with sufficient traveling experience, the weekend night markets have become too staged. They search for more local and mundane markets to construct their authentic local foodscape.

### 6.2. Theoretical Contributions

By proposing the typological framework and the dimensional construct of destination foodscape, this study contributes to an interdisciplinary conversation between tourism and food studies. Current food studies researching foodscape highlight that this notion helps tackle the complexity of the dynamic food–human–place nexus and understand the social and spatial disparities of the food-related phenomenon [8]. This study pays special attention to the tourism context, a temporary and unstable cross-cultural interface, which is relatively neglected by the mainstream food literature. We conceptualize the destination foodscape into a six-dimensional networked construct, through which we highlight the dynamics of this notion by integrating the tourist experiential dimension with the destination material and social-cultural dimensions. In this sense, we improve the dimensional construct proposed by Björk and Kauppinen-Räisänen [14] by emphasizing the complexity of the mixed tourist food experience and highlighting the networking of the dimensions rather than acting on their own in the construction of the destination foodscape.

In terms of the typological framework, previous literature has evolved from the novelty-familiarity binary structure [10,41,71], the core-periphery structure [9], the novelty–familiarity spectrum structure [13], and the two-dimensional matrix model [14] to interpret tourists’ food consumption. By pointing out the in-betweenness of tourists’ food consumption and the familiar-novel hybrid experiences, Lin et al. proposed that more empirical research should be conducted in various cross-cultural travel contexts to verify new food consumption practices [13]. However, Lin et al.’s model is still too ambiguous to generalize the multifarious food experiences and destination foodscapes [13]. Just as they state, the phenomenon in which tourists choose to eat in non-home country ethnic restaurants should also be noted. This study refines a coordinate framework consisting of two spectrum axes that crucially influence tourists’ familiar or exotic experiences, thus accommodating more diversified types of foodscapes such as the overseas ethnic foodscape and the globalized recreational foodscape. The globalized recreational foodscape needs more research attention, as it marks the vacation and leisure attributes of a destination along with the breeding of some creative subcultures. Compared with Björk and Kauppinen-Räisänen’s matrix model, we emphasize the “spectrum” rather than the “binary” thought in the dimension of serving locals/tourists. We also point out the effect of different cultural distances in classifying destination foodscapes.

### 6.3. Practical Implications

This study provides some practical implications. First, based on the typological framework proposed by this study, destination managers and marketers could generally rethink and plan the possible types of destination foodscapes to promote destination attractiveness. Second, some market research should be conducted to understand the motive and perspectival characteristics of the target tourist markets. Third, the destination food and place resources should be considered where relevant and planned in connection with the mixed familiar-novel motives of the target tourists to construct specific types of destination foodscapes. Finally, the marketing departments should also consider using the virtual tourism community to promote the construction of destination foodscapes.

### 6.4. Future Studies

Due to the time and accessibility limits, this study mainly chose Mafengwo as the platform to collect data, in which the sample is mainly concentrated in young and middle-aged groups. Future studies could be conducted in two directions. First, more comparative studies could be conducted among different tourist segments, such as female tourists, family tourists and elderly tourists, and different cross-cultural contexts, regarding the destination foodscape construction. Second, it will also benefit destination development by taking a diachronic examination of specific destination dining spaces regarding their evolution in the types of destination foodscape with the engagement of the primary tourist market.

## Figures and Tables

**Table 1:** A summary of different destination foodscapes.

Dimensions	Elements and Connotations	Globalized Recreational Foodscape	Staged Local Foodscape	Glocalized Foodscape	Authentic Local Foodscape	Overseas Ethnic Foodscape
Consumption grade	Price level and grade	Medium to high level of consumption	Medium to high level of consumption	Folk level of consumption	Folk level of consumption	Medium to high level of consumption
Physical environment	Functionality, location and decoration	Modern, creative and artistic cafeterias or dessert shops	Staging symbols of local representative culture, religion or environment	Standard decoration integrated with some local elements	Local markets or local, humble restaurants	Restaurants with strong ethnic and national symbols
Social interaction and ambiance	Tourists’ interaction with the locals, service personnel, other guests; or immersion within the social ambiance	Limited within ordering food with the service personnel, static gaze upon international tourists	Limited within ordering food with the service personnel, static gaze upon international tourists	Limited within ordering food with the service personnel, static gaze upon locals	Communicate with locals, immersion within the folklore, mundane life	Ordering food with the locals/service personnel; static gaze upon locals
Food and eating	ingredients, taste, healthy effect, appearance, hygiene, eating method/dining habits	Familiar dessert and drinks with creative and good-looking appearances, photo-taking	Local ingredients, Reformed taste	Standard food packaging and formula, with local special ingredients	Nonstandard hygienic conditions, local ingredients and authentic taste	Ethnic cuisine integrated with some local cooking style
Culture	various cultures in the spectrum of cultural distance	Global culture with local creative and arctic subculture	Local natural conditions, regional and historical culture	Global fast-food culture	Local folklore culture	Ethnic culture of different nations embedded in the destination
Tourist’s mixed experience	Familiarity- novelty spectrum	Experience within a familiar touristic clave with some exotic ambiance	Experience within a familiar environmental bubble with staged local culture	Experience within a familiar environmental bubble hybrid with some local exotics	Intense novelty within the authentic local	Intense familiarity with a small amount of locality/renewal of exotic experience

## Data Availability

All related data and methods are presented in this paper and the Appendix A. Additional inquiries should be addressed to the corresponding author.

## Appendix A

**Table A1:** Basic information of the travelogues in Mafengwo.

No.	Gender	Posted Time	Departure Time	Travel Days	Fellow Traveler
M01	Male	2019/1/10	2019/1/9	22	alone
M02	Female	2017/4/6	2017/3/10	6	alone
M03	Male	2012/9/22	-	4	-
M04	Male	2017/1/31	2016/12/23	8	couple
M05	Male	2018/3/19	2018/1/31	about 60	friends
M06	Male	2018/1/6	2017/12/21	7	friends
M07	Male	2017/4/10	2017/2/27	9	alone
M08	Female	2016/4/14	2016/1/29	16	friends
M09	Female	2016/10/22	2016/7/12	12	alone
M10	Female	2016/3/11	2016/11/8	6	couple
M11	Female	2021/12/2	2019/11/8	6	friends
M12	Female	2019/11/24	2019/10/2	6	couple
M13	Female	2021/12/12	2020/4/30	5	couple
M14	Female	2021/12/4	2016/2/8	8	families
M15	Male	2021/11/1	2016/8/10	16	couple
M16	Female	2020/12/6	2019/11/22	8	friends
M17	Female	2021/3/5	2017/6/27	15	friends
M18	Male	2021/6/16	2020/9/28	1	alone
M19	Female	2019/4/20	2019/4/11	4	friends
M20	Female	2019/5/24	2019/4/27	5	couple
M21	Female	2020/12/8	2019/7/28	6	friends
M22	Male	2021/1/31	2019/11/10	7	friends
M23	Female	2020/10/10	2020/1/16	13	families
M24	Male	2021/1/13	-	-	-
M25	Female	2017/8/22	/2017/05/01	9	friends
M26	Male	2017/4/17	2017/2/5	8	alone
M27	Female	2020/8/1	2019/7/19	7	friends
M28	Female	2020/7/14	2020/7/21	10	friends
M29	Female	2020/8/10	2019/9/11	7	families
M30	Female	2018/3/23	2017/8/27	64	friends
M31	Female	2016/10/23	2016/8/21	12	friends
M32	Male	2020/4/28	2017/11/16	9	families
M33	Female	2020/5/25	-	-	-
M34	Female	2017/11/12	2017/6/24	11	families
M35	Female	2012/12/25	2012/11/22	11	couple
M36	Female	2019/7/6	2019/6/23	7	couple
M37	Female	2013/12/26	2013/12/12	9	couple
M38	Female	2015/12/31	2015/4/26	9	alone
M39	Female	2016/6/18	2016/5/23	7	couple
M40	Female	2012/2/28	-	11	alone
M41	Male	2019/12/6	2019/8/7	6	families
M42	Female	2016/7/1	2016/5/7	8	couple
M43	Female	2014/6/20	2014/5/30	8	couple
M44	Female	2020/4/30	2017/1/30	10	couple
M45	Male	2016/8/24	2016/4/30	8	couple
M46	Male	2020/4/20	-	-	alone
M47	Female	2020/4/30	2019/9/28	6	couple
M48	Female	2020/4/13	2019/9/18	6	friends
M49	Female	2020/3/7	2018/9/24	7	couple
M50	Female	2020/3/27	2020/1/16	7	-
M51	Female	2020/4/8	2019/12/10	50	alone
M52	Female	2020/2/23	2019/12/6	8	couple
M53	Female	2013/1/1	-	-	-
M54	Male	2020/5/4	2019/8/1	7	alone
M55	Male	2021/11/10	-	-	-
M56	Male	2019/10/19	-	-	-
M57	Male	2020/7/4	2015/7/27	7	families
M58	Female	2020/8/4	-	-	-
M59	Female	2019/10/19	2019/11/5	5	friends
M60	Male	2019/12/1	-	-	-
M61	Female	2020/10/25	2017/7/9	6	friends
M62	Female	2019/1/31	2019/1/30	7	couple
M63	Female	2019/2/11	2019/1/21	8	couple
M64	Female	2021/2/25	2018/10/1	6	friends
M65	Female	2018/8/31	2018/8/3	20	families
M66	Male	2019/2/11	2019/1/12	10	families
M67	Male	2019/11/10	2019/11/6	5	colleague
M68	Male	2019/7/12	2019/6/18	3	friends
M69	Female	2018/11/22	2018/11/10	8	friends
M70	Female	2018/1/25	2018/1/3	5	-
M71	Female	2018/5/19	2018/5/10	1	friends
M72	Female	2019/3/28	2019/3/23	1	couple
M73	Male	2018/8/4	2018/7/9	6	friends
M74	Female	2017/8/5	-	-	-
M75	Female	2017/11/8	2017/10/28	8	friends
M76	Male	2018/3/4	2015/3/4	10	friends
M77	Male	2019/2/22	2019/2/3	4	couple
M78	Male	2017/4/16	2016/3/26	18	alone
M79	Female	2017/12/3	2017/10/20	10	families
M80	Female	2020/2/11	2019/12/27	6	friends
M81	Female	2019/1/25	2019/1/3	8	friends
M82	Female	2018/10/9	2018/9/25	5	friends
M83	Female	2019/7/24	2018/9/8	8	alone
M84	Female	2019/12/7	2019/11/22	5	families
M85	Female	2015/6/22	2014/7/16	16	families
M86	Female	2012/9/24	2012/9/14	11	-

**Table A2:** Profiles of Informants interviewed online.

No.	No. in Travelogues	Gender	Age	Origin (City, Province)	Profession	Interview Platform	Length of Interview
I01	M36	Female	34	Yantai, Shandong	Employee of public institutions	Tencent meeting	66 min
I02	M78	Male	66	Chengdu, Sichuan	College professor	Tencent meeting	48 min
I03	M40	Female	32	Shenzhen, Guangdong	Securities practitioner	Tencent meeting	49 min
I04	M71	Female	35	Chenzhou, Hunan	Staff of tourism enterprise	Wechat	About 40 min
